# The Catalytic Degradation of Waste PU and the Preparation of Recycled Materials

**DOI:** 10.3390/polym16243581

**Published:** 2024-12-21

**Authors:** Xiaohua Gu, Jiahao Xu, Shangwen Zhu, Qinglong Zhao, Shaochun Sun, Yanxun Zhang, Qingyong Su, Canyan Long

**Affiliations:** 1 School of Energy and Building Environment, Guilin University of Aerospace Technology, Guilin 541004, China; xujiaohao0711@163.com (J.X.); 2022043@guat.edu.cn (S.Z.); 2023060120234@stu.guat.edu.cn (S.S.); 2021008@guat.edu.cn (Y.Z.); sqy@guat.edu.cn (Q.S.); longcanyan@guat.edu.cn (C.L.); 2 Qiqihar School of Materials Science and Engineering, Qiqihar University, Qiqihar 161006, China; 3 Guangxi Key Laboratory of Green Building Materials and Construction Industrialization, Guilin University of Technology, Guilin 541004, China; 4 University Engineering Research Center of Green Upgrade Key Technology for Energy Industry, Guilin 541004, China; 5 Guangxi Key Laboratory of Special Engineering Equipment and Control, Guilin University of Aerospace Technology, Guilin 541004, China; 6 College of Civil Engineering and Architecture, Northeast Petroleum University, Daqing 163318, China; 238003050924@stu.nepu.edu.cn

**Keywords:** waste polyurethane, PN catalyst, alcoholysis, recycled materials

## Abstract

In this paper, we investigated the efficient metal-free phosphorus–nitrogen (PN) catalyst and used the PN catalyst to degrade waste PU with two-component binary mixed alcohols as the alcohol solvent. We examined the effects of reaction temperature, time, and other factors on the hydroxyl value and viscosity of the degradation products; focused on the changing rules of the hydroxyl value, viscosity, and molecular weight of polyols recovered from degradation products with different dosages of the metal-free PN catalyst; and determined the optimal experimental conditions of reaction temperature 180 °C, reaction time 3 h, and PN dosage 0.08%. The optimal experimental conditions were 180 °C, 3 h reaction time, and 0.08% PN dosage, the obtained polyol viscosity was 3716 mPa·s, the hydroxyl value was 409.2 mgKOH/g, and the number average molecular weight was 2616. The FTIR, ^1^H, NMR, and other tests showed that the waste urethanes were degraded into oligomers successfully, the recycled polyether polyols were obtained, and a series of recycled polyurethanes with different substitution ratios were then prepared. A series of recycled polyurethane materials with different substitution rates were then prepared and characterized by FTIR, SEM, compression strength, and thermal conductivity tests, which showed that the recycled polyurethane foams had good physical properties such as compression strength and apparent density, and the SEM test at a 20% substitution rate showed that the recycled polyol helped to improve the structure of the blisters.

## 1. Introduction

Due to the wide application of polyurethane (PU) in various industries such as construction, automotive, and consumer goods, its global production and consumption have seen significant growth. However, the widespread use of polyurethanes has also led to a large accumulation of polyurethane waste, which poses great environmental and economic challenges. It is estimated that millions of tons of polyurethane waste are generated each year, reaching around 25 million tons worldwide, and this figure is expected to increase with the growth of polyurethane production. Waste polyurethane is an insoluble and insoluble thermosetting polymer material, is difficult to deal with, unreasonable recycling and degradation may cause serious environmental pollution to soil and water, but it also may destroy the balance of the ecosystem, and long-term storage will increase the burden on the environment.

In terms of environmental impact, the disposal of polyurethane waste in landfills and in the natural environment can have harmful effects on ecosystems. It is well known that polyurethane materials are resistant to biodegradation and therefore will persist in the environment for a long time. This persistence not only takes up valuable landfill space, but also causes soil and water pollution through the leaching of toxic substances. In addition, the production of polyurethane is associated with high energy consumption and greenhouse gas emissions, further exacerbating climate change. In terms of economic impact, the economic impact of polyurethane waste is equally worrying. The disposal and management of polyurethane waste requires significant financial resources, and the potential loss of value of discarded polyurethane material means a missed opportunity for economic gain. The recycling and recovery of polyurethane waste can reduce these costs by reducing the need for raw material extraction and reducing waste disposal costs.

Although there are more research reports on the recycling of waste PU rigid foam in China, the recycling of waste materials can effectively reduce pollution, protect the environment, and save resources. From the economic point of view, for the recycling of waste PU rigid foam materials, direct recycling is the best, but the product performance is poor. From the aspect of product performance, it is still better to use a hydrolyzing, alcohol decomposition, alkali decomposition type of chemical method, and the difficulty of the chemical method of recycling is the selection of catalysts and other catalysts in the degradation process. Domestic and foreign research on the degradation of waste polyurethane rarely mentioned the influence of catalysts as the traditional catalyst degradation efficiency is low, so it is very important to study new efficient catalysts for the degradation of waste polyurethane. Most of the current research on the degradation and recycling of waste polyurethane is to use alkali metal hydroxides and their salts as catalysts for the degradation reaction; the metal ions will remain in the degradation product of recycled polyol, which is easy to cause the gel phenomenon [1,2]. Therefore, the content of alkali metal ions must not be greater than 1 × 10^−5^. Moreover, the alkali metal hydroxide catalyst in the reaction system is difficult to precipitate after the reaction, and even if precipitated, it is difficult to separate [3,4]. Therefore, in this paper, the recycling catalyst of polyurethane waste is explored, the waste PU hard foam is used as the raw material, and the PN catalyst, which does not contain metal ions, is used for the degradation of waste polyurethane and recycling to obtain the regenerated PU hard foam.

In this study, in the presence of small molecule alcohols such as ethylene glycol (EG), ethanolamine (MEA), and a metal-ion-free PN catalyst, the urethane bond in waste polyurethane (WPUF) was broken and replaced by a relatively short alcohol chain, which resulted in the formation of polyurethane’s degradation product, recycled polyol [5,6]. The alcoholysis products were also tested for infrared spectroscopy, viscosity, and hydroxyl value so as to obtain efficient catalytic degradation catalysts for the effective degradation of waste polyurethane and to optimize the best degradation method. Then, the alcoholysis products were used to prepare polyurethane materials by replacing polyether polyol, exploring the effect of the amount of recycled polyol substitution on the performance of recycled polyurethane, providing a more economically feasible way to synthesize polyurethane without significantly reducing the performance of the materials, and realizing green environmental protection, pollution reduction and carbon reduction, circular economy.

## 2. Experimental Section

### 2.1. Main Raw Materials and Reagents

Waste polyurethane rigid foam; PN catalyst, Sinopharm Group Chemical Reagent Co., Ltd., Shanghai China; Ethylene glycol, Tianjin Kaitong Chemical Reagent Co. Ltd., Tianjin, China, analytical pure (AR), purity is 99.5%; Ethanolamine, Tianjin Kaitong Chemical Reagent Co., Ltd., Tianjin, China, analytically pure (AR), purity is 99%; Triethanolamine, Tianjin Kaitong Chemical Reagent Co., Ltd., Tianjin, China, analytical pure (AR); Dibutyl dilaurate, Tianjin Kaitong Chemical Reagent Co, Tianjin, China.

### 2.2. Major Equipment and Instruments

Degradation Reactor, Gongyi Iohua Instrument Co., Ltd., Gongyi, China; Fourier Transform Infrared Spectroscopy (FTIR), Spectrum-one, PE, USA; Circulating Water Vacuum Pump, SHZ-IIIA, Gongyi Iohua Instrument Co., Ltd., Gongyi, China; Gel Gas Chromatograph, OPTILAB-F, Wyatt, MI, USA; Scanning Electron Microscope, S-3400, Hitachi, Tokyo, Japan; Nuclear Magnetic Resonance Instrument, AVANCEAV400MHz, Bruker, Karlsruhe, Germany. Thermal Conductivity Tester, DRE-III, Xiangtan Xiangyi Instrument Co., Xiangtan, China.

### 2.3. Experimental Methods

#### 2.3.1. Degradation of Used Polyurethane

EG and MEA in the mass ratio of 6:4 were added to a three-neck reactor equipped with a reflux condenser, and different ratios of PN (P(N(CH_3_)_2_)_3_NH) catalyst and 100 g of WPUF pulverized to 0.2–1 mm were added to the reaction system. The mass ratio (i.e., solid–liquid ratio) of WPUF to the mixed alcohol degradation agent, ethylene glycol (EG), and methyl alcohol amine (MEA) was 1:1.5. The reaction was heated and stirred at a predetermined temperature, and the reaction was carried out with stirring. The degradation product recycled polyol RP was obtained after the reaction period [7,8].

#### 2.3.2. Preparation of Recycled Polyurethane Materials

Recycled polyurethane (RPU) was prepared by a one-step method, and the formula is shown in Table 1. First, a preset amount of polyether polyol 4110, self-made recycled polyol RP-0.08, dimethylsilicone oil, monofluorodichloroethane, triethanolamine, and dibutyltin dilaurate were added to the plastic cup, and were mixed at a room temperature of about 20–25 °C and humidity below 50% by mechanical stirring at 500 rpm for 30 s to mix evenly. PAPI was then added to the polyol mixture and mixed at 1000 rpm until foamed. The RPU was tested after being placed at a room temperature of about 20–25°C and humidity below 50% for 24 h. The regenerated polyurethane foam prepared with an RP substitution rate of 10%, 20%, 30%, and 40% was labeled as RPU-0 (that is, 100% 4110 polyurethane), RPU-10, RPU-20, RPU-30, and RPU-40, respectively.

**Figure 1:**
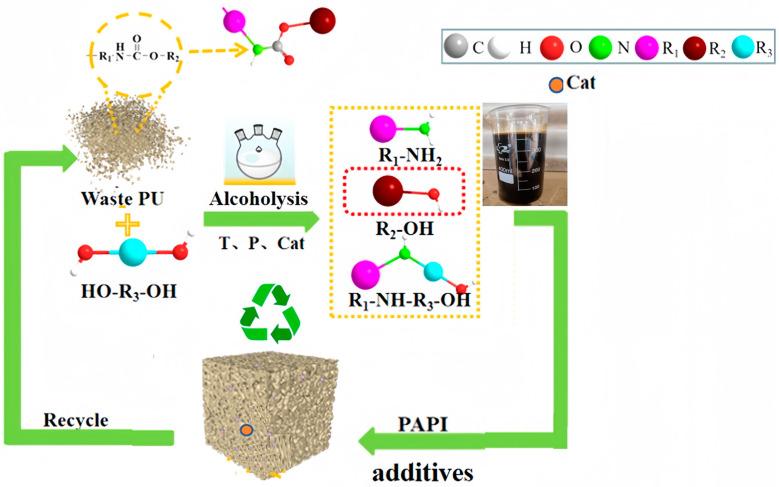
Degradation process flow diagram for polyurethane degradation with PN catalysts.

**Figure 2:**
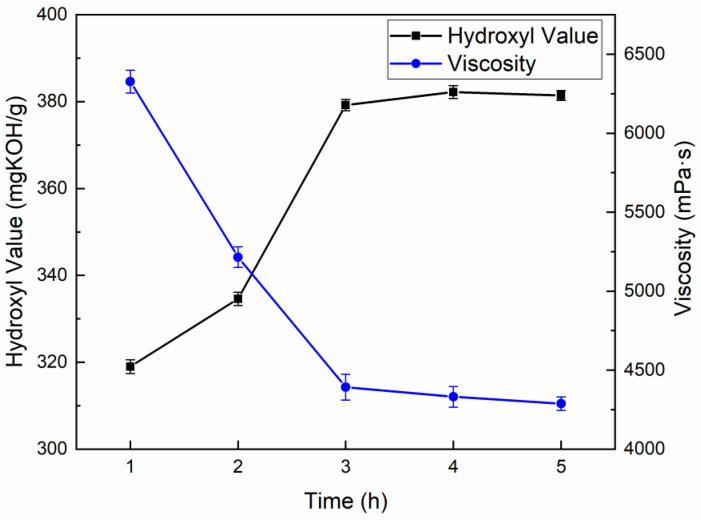
The hydroxyl value and viscosity of recovered polyols at different reaction times.

The cyclic process flow of catalytic degradation of waste PU by PN catalyst and preparation of recycled materials is shown in Figure 1, R1 and R2 are shown in Figure 2, and R3 is the mixed alcohol EG and MEA.

### 2.4. Performance Testing and Characterization

#### 2.4.1. Fourier Transform Infrared Spectrometer (FTIR)

For the performance test and characterization of the recovered polyols, the chemical structure of the recovered polyols was determined by the Spectrum One Fourier transform infrared spectrometer (FTIR) of the PE Company in the United States, and the samples were tested in a dry environment at room temperature using ATR accessories. The scanning range is 4000~500 cm^−1^, and the scanning resolution is less than 0.09 cm^−1^.

For the performance test and characterization of the recycled polyurethane, the chemical structure of the recycled polyurethane sample was determined by the Spectrum One Fourier transform infrared spectrometer (FTIR) of the PE Company in the United States. The dried polyurethane sample was prepared by KBr lamination in the dry environment at room temperature, and the ATR attachment was used for testing. The scanning range is 4000~500 cm^−1^, and the scanning resolution is less than 0.09 cm^−1^ [9].

First, the sample needs to be filtered and purified, and the sample is pressed into thin sheets. The Fourier transform infrared spectrometer is calibrated, the background spectrum is recorded before placing the sample, and the prepared sample is placed on the FTIR sample holder, ensuring that the sample is flat and perpendicular to the beam. The resolution and scanning range were set, and the FTIR software was used to process the collected data and analyze the FTIR spectra. The characteristic peaks were identified and the chemical bonds were determined, the functional groups and chemical compositions in the recovered polyols were determined according to the characteristic peaks in the FTIR spectra, and the purity of the recovered polyols was evaluated by analyzing the characteristic peaks of impurities in the spectra.

#### 2.4.2. Nuclear Magnetic Resonance (NMR)

The AVANCEAV400MHz NMR instrument of Bruker, Germany, was used to ensure the preheating and stability of the NMR instrument. Deuterium dimethyl sulphonide (DMSO-d_6_) was used as the solvent and tetramethylsilane (TMS) was used as the internal standard to test the recovered polyols. The dissolved sample was loaded into the NMR tube, the NMR parameters were set, and the NMR data were collected. The chemical structure of the degradation products was determined according to the characteristic peaks in the NMR spectra. The purity of the degradation products was evaluated by analyzing the characteristic peaks of impurities in the spectra.

#### 2.4.3. Viscosity Test

The appropriate amount of degradation product is placed in a beaker, and the sample does not contain any visible impurities and bubbles. NDJ-5S rotary viscometer was used to test the viscosity of the recovered polyol RP at 25 °C after calibration. The viscometer was placed in the prepared sample solution, the viscometer was started, the torque and speed were recorded, and, thus, the viscosity value of the sample was obtained.

#### 2.4.4. Hydroxyl Value Test

According to the international standard, an appropriate amount of oligomer polyol was placed in a 10 mL conical bottle, and the hydroxyl value of the recovered polyol was determined by the ester anhydride method and pyridine. The sample solution was placed under the burette and the standard alkali solution was slowly added until the indicator color changed, and the volume of the standard alkali solution consumed when the neutralization end point was reached was recorded. According to the volume and concentration of the standard alkali solution consumed, the content of hydroxyl in the sample was calculated.

#### 2.4.5. Gel Permeation Chromatography (GPC)

Molecular weight distribution is a scientific measure of the chain length and dispersion in the molecular chain structure of a polymer [10]. The molecular weight distribution directly affects the physical and chemical properties of polyols recovered from degradation products, and also directly affects the application processing properties of downstream applications such as polyurethane. The recovered polyols were tested by gel permeation chromatography on Series 200 with polystyrene as the standard sample and tetrahydrofuran as the mobile phase. Column types include PLgel 10 µm Mixed-B, which is used to separate polyurethane degradation products of different molecular weights. PLgel 5 µm Mixed-C was used for the further refinement of molecular weight distribution analysis. The flow rate of the mobile phase was 1.0 mL/min, the detector was a differential refraction detector, and the temperature was 35°C.

#### 2.4.6. Compression Strength Test

According to the international standard, the compression strength of recycled polyurethane was tested by an HKW 50 kn universal material testing machine. The size of the selected sample was 50 × 50 × 50 mm. The cross beam displacement speed of the instrument was 20 mm/min, and the samples were tested, respectively. Based on the recorded force and displacement data, the stress and strain were calculated and the compressive strength was determined from the stress–strain curve. Each sample was measured 5 times, and the average value was taken.

#### 2.4.7. Apparent Density Test

The apparent density of RPU was measured according to the international standard. The mass of the sample was measured accurately by electronic balance, the size of the sample was measured by spiral micrometer, and the volume of the sample was calculated according to the measured size. The apparent density of 5 samples of each regenerated polyurethane sample was measured and the average value was calculated.

#### 2.4.8. Thermal Conductivity Test

Thermal conductivity is the physical quantity to evaluate the thermodynamic properties of materials, which is of great significance as a main evaluation basis for the application of thermal insulation materials in construction, cold chain, home appliances, and other industries. The lower the thermal conductivity, the slower the heat transfer inside the material, and the better the thermal insulation performance of the material [11]. A thermal conductivity tester was used to measure the thermal conductivity of polyurethane samples according to the international standard. The sample is processed into a standard size sample to meet the requirements of the thermal conductivity test. The thermal conductivity tester was calibrated, the test parameters were set, and the prepared sample was placed at the test position of the measuring instrument to ensure that the sample was smooth and perpendicular to the direction of heat flow. The thermal conductivity measuring instrument was started and tested according to the set parameters. Data such as temperature change, heat flow, and time of the sample were recorded during the test. According to the recorded data and the principle of the test method, the thermal conductivity of the sample was calculated.

#### 2.4.9. Scanning Electron Microscope (SEM)

The recycled polyurethane slice samples of different components were selected to ensure that the surface of the samples was clean and free of oil, and the samples were placed on the sample table of the PVD equipment. The PVD device was started, vacuum pumping was performed first, the gold was installed on the evaporation source, the evaporation source was started, and the gold was evaporated and deposited on the sample surface. During deposition, the sample table was continuously rotated to ensure a uniform distribution of the coating. The deposition process was monitored in real time to ensure the uniformity and quality of the coating. The magnification was 20.0 kv × 60, and the pore morphology and skeleton structure of each sample were observed.

#### 2.4.10. Heat Loss Analysis

Thermogravimetric analysis is an important technique used to study the thermal stability and composition of materials. The polyurethane foam sample was cut and pulverized into suitable small pieces, while ensuring that the sample was dry to avoid moisture interference with the thermogravimetric analysis results. The thermogravimetric analysis was carried out by using a thermogravimetric analyzer in a nitrogen atmosphere and using 30 °C as the heating rate to increase the test sample from a room temperature of 25 °C to 600 °C. The gas flow level was 50 mL/min, the carrier gas was air, and alumina was used as the reference object. The initial mass of the sample was recorded and the prepared sample was placed in the TGA sample tray to ensure uniform sample distribution. The TGA instrument was started, it was tested according to the set temperature program, the mass change, temperature, and time of the sample were recorded during the test. According to the recorded data, the mass loss percentage of the sample was calculated, the mass loss curve was analyzed, the thermal stability parameters of the material were determined, and the thermal stability and degradation behavior of the regenerated polyurethane foam were evaluated according to the results of thermogravimetric analysis.

## 3. Results and Discussion

### 3.1. Recovered Polyol (RP) Test Results and Discussion

#### 3.1.1. Selection of Degradation Time

In this paper, EG and MEA were used as alcohololytics in a mass ratio of 6:4, with a solid–liquid ratio of 1: 1.5 and a PN catalyst dosage of 0.08% (based on the total mass of alcohololytics and WPUF). The reaction times of 1.0, 2.0, 3.0, 4.0, and 5.0 h were used as the experimental factor when the reaction temperature was 180 °C. The hydroxyl values and viscosities of the recovered polyols at different reaction times are shown in Figure 2.

As can be seen from Figure 2, with the increase in reaction time, the hydroxyl value of the recycled polyol gradually increased to basically unchanged, and the viscosity gradually decreased. When the reaction time is between 1.0 and 3.0 h, the hydroxyl value of the recycled polyol increases sharply and the viscosity decreases significantly, indicating that the degradation reaction of the waste polyurethane is intense, and the urethane bond is broken and replaced by alcohol hydroxyl, generating a large number of hydroxyl compounds. After the reaction time exceeded 3.0 h, the hydroxyl value and viscosity did not change much and tended to stabilize, indicating that the reaction time that exceeded 3 h had less influence on the two, and the reaction time was determined to be 3 h [12,13].

#### 3.1.2. Selection of Reaction Temperature

**Figure 3:**
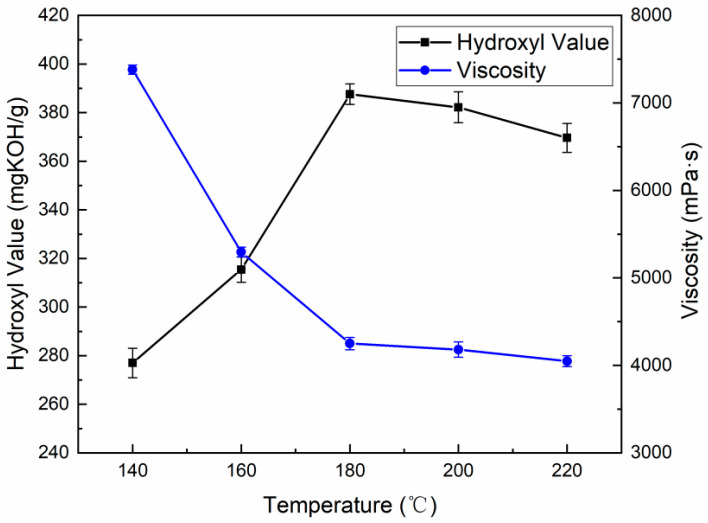
The hydroxyl value and viscosity of recovered polyols at different reaction temperatures.

In this experiment, EG and MEA were used as the alcoholysis agent in a mass ratio of 6:4; the solid–liquid ratio was 1:1.5 and the PN catalyst dosage was 0.08% (based on the total mass of alcoholysis agent and WPUF). When the degradation time was 3 h, the reaction temperatures of 140, 160, 180, 200, and 220 °C were used as experimental factors. The hydroxyl values and viscosities of the recovered polyols at different reaction temperatures are shown in Figure 3.

The hydroxyl value of the recovered polyol increased first with the increase in the reaction temperature, and reached the maximum value of 387.6 mgKOH/g at 180 °C [14]. As the temperature continued to increase, the hydroxyl value decreased slightly, and the viscosity did not change much [15]. The degradation of WPUF is a heat-absorbing process, so the increase in the reaction temperature can speed up the movement speed of EG and MEA molecules, which is favorable for the molecular contact between the molecules of the alcohololytic agent and the molecules of WPUF, and promotes the WPUF molecular contact between the alcohol solvent molecules and WPUF molecules, promoting the degradation reaction of WPUF in a positive direction. However, too high a reaction temperature will promote the oxidation, etherification, and other side reactions of the alcohololytic agent, and also cause it to volatilize in large quantities, resulting in a reduction in reactants, which is not conducive to the positive reaction. At the same time, the degradation product of recycled polyol is also prone to a decarboxylation reaction, thus losing the reaction-active center [16]. Comprehensive consideration can determine that the appropriate reaction temperature is 180 °C.

#### 3.1.3. Selection of PN Catalyst Dosage

**Figure 4:**
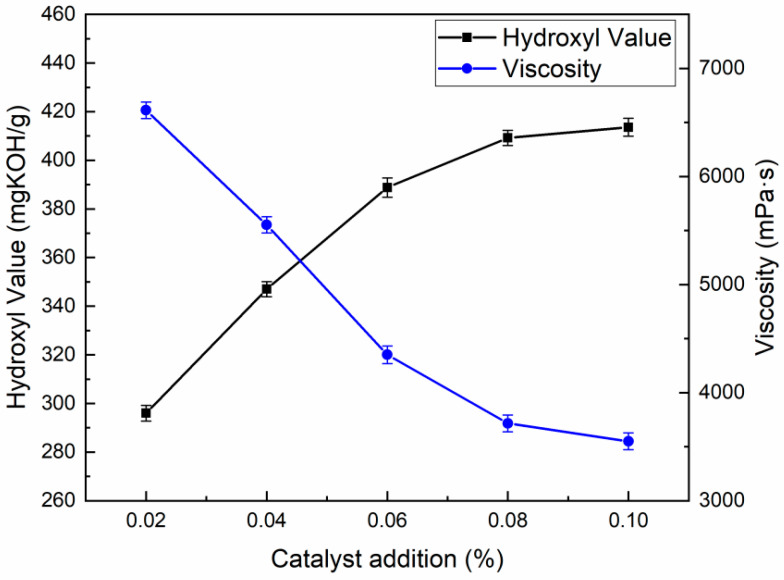
Hydroxyl value and viscosity of recycled polyols at different catalyst additions.

Catalysts play an important role in the degradation of waste polyurethanes, and its dosage significantly affects the degradation effect, determines the usability of the degradation product of recycled polyols, and also affects the cost of industrial applications [17]. In this experiment, EG and MEA were used as alcohololytics in the mass ratio of 6:4 with a solid–liquid ratio of 1:1.5, and the reaction was carried out at 180 °C for 3 h. The recovered polyols obtained by using the amount of metal-free ionic PN catalysts of 0.02%, 0.04%, 0.06%, 0.08%, and 0.10% (based on the total mass of the alcohololytics and the WPUF) were, in the experimental factors, numbered in order as follows: RP-0.02, RP-0.04, RP-0.06, RP-0.08, and RP-0.10, and their hydroxyl values and viscosities are shown in Figure 4.

As can be seen from Figure 4, the hydroxyl value of the degradation product of recovered polyol increased with the increase in the PN catalyst dosage, while the viscosity gradually decreased. When the amount of PN catalyst was in the range of 0.02–0.08%, the hydroxyl value of the recovered polyol increased sharply and the viscosity decreased greatly, indicating that the amount of catalyst had a significant effect on the viscosity and hydroxyl value of the product. After that, the viscosity and hydroxyl value of the recovered polyol changed less, and the effect of the catalyst on the two gradually weakened. Therefore, it was initially determined that the amount of PN catalyst was 0.08%, at which time the viscosity of the recovered polyol was 3716 mPa·s and the hydroxyl value was 409.2 mgKOH/g.

#### 3.1.4. GPC Analysis of Recovered Polyols

**Figure 5:**
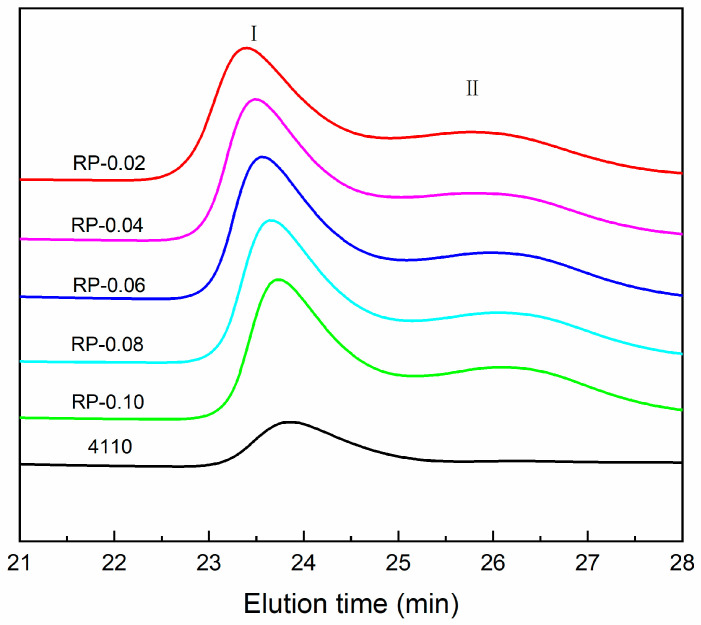
GPC curve of polyether 4110 and recovered polyols at different catalyst dosages.

The GPC test results of the recycled polyol and polyether 4110 are shown in Figure 5 and Table 2. The polyurethane chain is decomposed into low-molecular-weight chain segments under the action of the alcohololytic agent and PN catalyst. Peak I in Figure 5 is attributed to the recycled polyol, whose elution time is similar to that of polyether polyol 4110, which is an oligomer. Peak II represents the small molecule aromatic by-products formed when the hard segments of polyurethanes are replaced by alcoholysis agents during the transesterification process [18]. The number of the average molecular weight (Mn) of the degradation products of recovered polyols ranged from 4804 to 2465, which gradually decreased with the increase in PN catalyst dosage, and the elution time was gradually prolonged. When the catalyst dosage exceeded 0.08%, the recovered polyol Mn and PDI did not change much.

#### 3.1.5. Infrared Spectroscopy of Recovered Polyols

**Figure 6:**
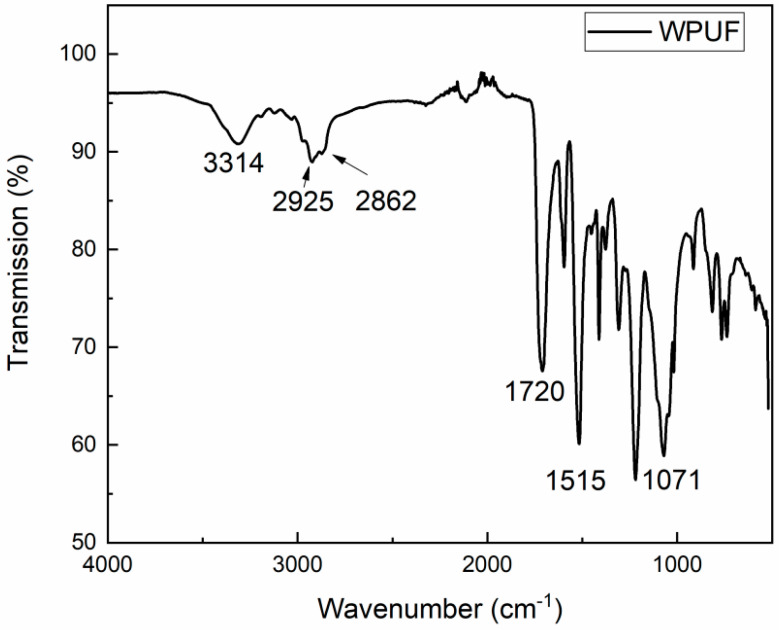
Infrared spectrum of WPUF.

The infrared spectrum of used polyurethane rigid foam WPUF is shown in Figure 6. The spectrum shows some main peaks of WPUF: C-O stretching vibration peak (1071 cm^−1^), -Ar (1515 cm^−1^), -NHCO- (amide I band, 1720 cm^−1^), CH_2_ stretching vibration peaks (about 2925 cm^−1^ and 2862 cm^−1^), and -NH stretching vibration peaks (ca. 3314 cm^−1^) [19]. It can be seen that WPUF is a polyether-type polyurethane.

**Figure 7:**
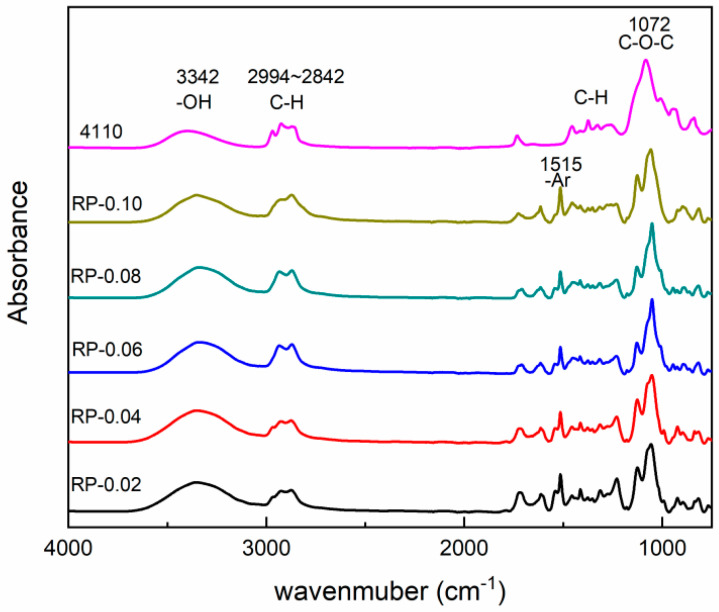
Infrared spectra of RP and polyether 4110.

As can be seen from Figure 7, the recovered polyol is basically similar to commercial polyether 4110 in terms of characteristic peak patterns. There is a clear and strong absorption peak at 3342 cm^−1^, which is attributed to the alcohol hydroxyl group stretching vibration peak [20]. The characteristic absorption peaks at 2994–2842 cm^−1^ are the C-H stretching vibrations in CH, CH_2_,CH_3_ [21]. Compared with recycled polyol, the absorption peak of polyether 4110 is stronger here, which may be caused by the presence of more long-chain structures in polyether 4110. The absorption peak at the peak near 1515 cm^−1^ is the absorption peak of the benzene ring, which does not exist in 4110, i.e., it does not contain the benzene ring, which suggests that the use of recycled polyols to prepare recycled polyurethanes can introduce rigid groups into the material’s matrix, which is conducive to enhancing its mechanical properties. There are absorption peaks at 1454 cm^−1^ and 1375 cm^−1^, which are characteristic of C-H bending vibration in polyol chains [22]. The absorption peak at 1072 cm^−1^ corresponds to the C-O-C vibration. The results indicate that a large number of carbamate bonds in WPUF were broken and replaced by alcohol hydroxyl groups in the presence of alcohololytic agents and PN catalysts, and were degraded to recycled polyols containing ether bonds.

#### 3.1.6. NMR Analysis of Recycled Polyols

**Figure 8:**
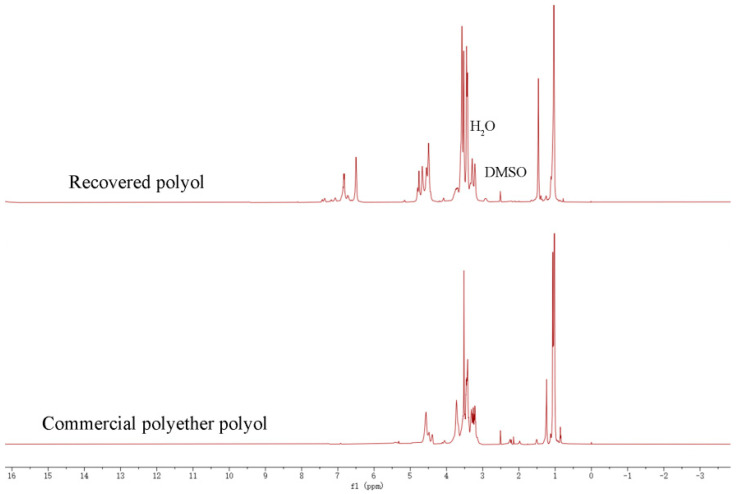
^1^H NMR spectra of recovered polyols and commercial polyether polyols.

The recycled polyol RP−0.08 and commercial polyether 4110 were characterized by NMR hydrogen spectroscopy, and the spectra are shown in Figure 8. The proton peaks at 1.03−1.46 ppm are for methyl groups, and the absorption peaks at 3.32−3.64 ppm are attributed to methylene groups affected by hydroxyl groups and methylene groups attached to ether bonds [23]. The chemical shift at around 3.51−4.50 ppm is the proton peak of the terminal hydroxyl group. The proton peak at 6.50−6.86 ppm is the absorption of hydrogen atoms on the benzene ring [24], which originates from the hard segment of the polyurethane. The comparison shows that the ^1^H NMR spectra of the degradation product and the commercial polyether polyol are similar, which proves that the regenerated polyether polyol was successfully prepared.

#### 3.1.7. Mechanism of Degradation of Waste Polyurethane by PN Catalyst

**Figure 9:**
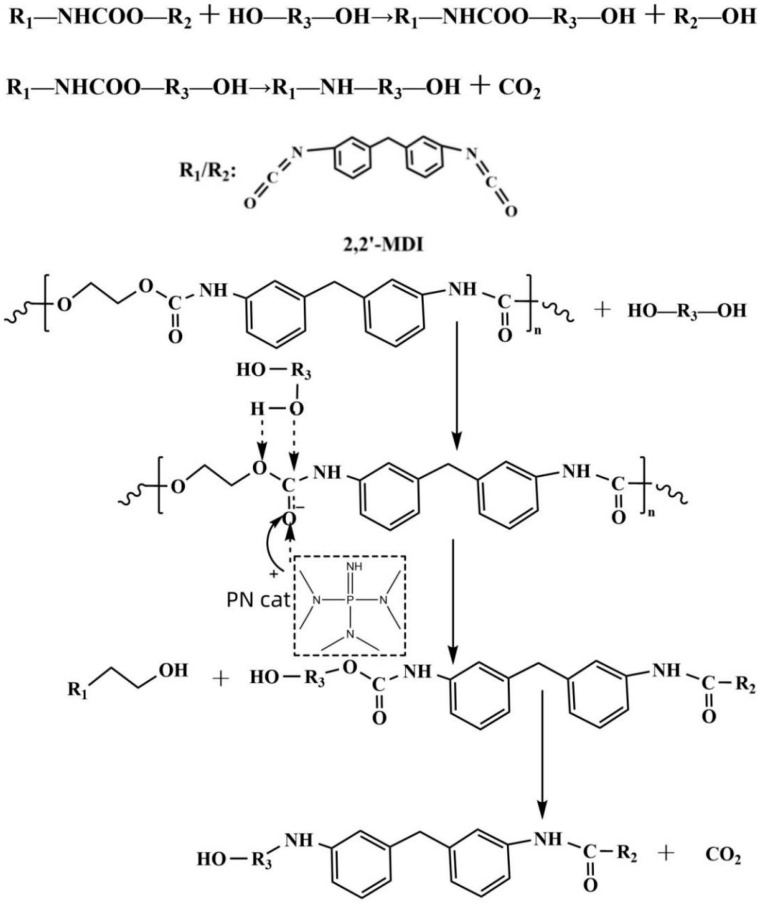
Schematic diagram of the mechanism of the catalytic degradation of waste polyurethane by PN catalysts.

Polyurethanes consist of carbamate links, which are polymeric polymers formed by the polyaddition reaction of diisocyanates and diols or polyols [25,26,27]. The mechanism of degradation of waste polyurethane involves several chemical reaction steps that work together to achieve the degradation of polyurethane. The principle of the PN catalyst to catalyze polyurethane is a relatively complex process. Nitrogen atoms and phosphorus atoms in the PN catalyst act as nucleophilic reagents, and nucleophilic addition and elimination reactions occur. The PN catalyst has strong alkalinity and high nucleophilicity, making it an effective catalyst. Under the action of the PN catalyst, carbonyl carbon atoms in a carbamate bond are attacked by the nucleophile, and a nucleophilic addition reaction occurs. The first is the activation of the PN catalyst. The catalyst forms coordination bonds with the carbonyl group (C=O) in polyurethane through the lone pair of electrons on its nitrogen atom. The coordination between the PN catalyst and the carbonyl group reduces the electron density of the carbonyl group and makes it more vulnerable to nucleophilic attack, thus activating the carbonyl group. Because the activated carbonyl group is more susceptible to attack by the alcohols of the nucleophile alcohololysis agent, this nucleophilic attack can lead to the transfer of electrons and the breaking and rearrangement of urethane bonds [28]. During the nucleophilic attack, a transition-state intermediate is formed with the phosphorus–nitrogen bond as the active center, in which the catalyst interacts with both the broken and unbroken parts of the carbonyl group of the carbamate bond. Subsequently, by eliminating the reaction, resulting in the break of the carbamate bond, the catalyst forms a new chemical bond with the group of the broken carbamate bond, forming the product of the hydroxyl group as the end group of the regenerated polyether polyol. The degradation reaction mechanism of polyurethane degraded by the PN catalyst is shown in Figure 9, in which R3 is mixed alcohol EG and MEA [29,30,31,32].

### 3.2. Test Results and Discussion of Recycled Polyurethane

#### 3.2.1. Infrared Analysis of Recycled Polyurethane

**Figure 10:**
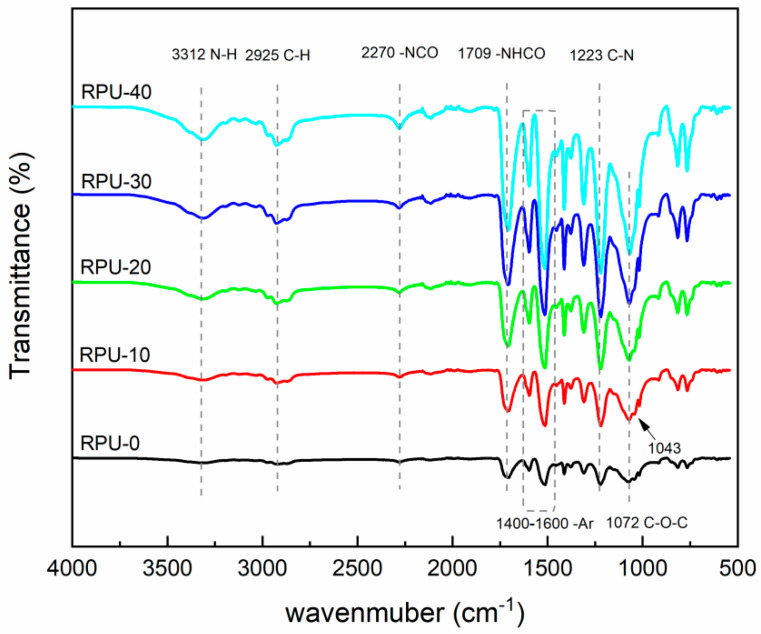
Infrared spectra of RPU with different recovery polyol substitution rates.

The IR spectra of the recycled polyurethane foams prepared from the recycled polyol RP-0.08 obtained at 0.08% PN dosage with different substitution rates are shown in Figure 10. All the foam samples showed similar absorption vibration peaks. The broad peak near 3312 cm^−1^ is characteristic of the stretching vibration signal of N-H, and the absorption peak at 1709 cm^−1^ is that of -NHC=O [33]. The stretching vibration peak of -C-H is near 2925 cm^−1^ [34], and there is a strong absorption peak at 1223 cm^−1^ attributed to the C-N in the carbamate bonding stretching vibration; 1400–1600 cm^−1^ is the vibrational characteristic peak of the aromatic skeleton. The red spectrogram of the RPU-0 sample has a very small peak at 2270 cm^−1^, which indicates that the polyol basically reacts completely with the isocyanate, and there is almost no residual -N=C=O. As the substitution rate of the recycled polyol increases, the absorption peak of -N=C=O gradually strengthens, which indicates that the reactivity of the recycled polyol is weaker than that of polyether 4110, and free isocyanate exists in the recycled polyurethane foam. The peak at 1072 cm^−1^ is attributed to the stretching vibration of the ether bond [35]. The peak at 1043 cm^−1^ corresponds to the characteristic absorption peak of -C-O- in the urethane ester [36]. It indicates that the presence of urea groups in the synthesized polyurethanes is conducive to improve the compressive strength of the recycled polyurethanes. The above analysis shows that the recycled polyurethane materials were successfully prepared by replacing polyether polyol 4110 with different amounts of recycled polyol.

#### 3.2.2. Compressive Strength and Apparent Density Analysis of Recycled Polyurethane

**Figure 11:**
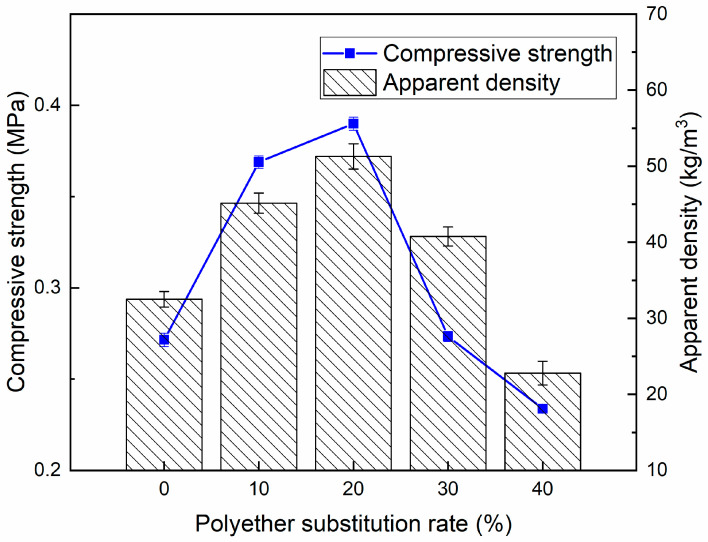
Compressive strength and apparent density of RPU at different recycled polyol substitution rates.

The compressive strength and apparent density of the recycled polyurethane foams prepared from recycled polyol RP-0.08 at different substitution rates are shown in Figure 11. The compressive strength of the recycled polyurethane material first increased and then decreased with the increase in substitution rate, reaching a maximum value of 0.390 MPa at a 20% substitution rate, which was 43.38% higher than that of pure petroleum-based polyurethane with a 0% substitution rate (0.272 MPa). This may be attributed to the fuller reaction between the blended polyol and the isocyanate when the substitution rate is small and the introduction of a large amount of aromatic structures derived from the isocyanate into the recycled polyurethane foam in the recycled polyol, with an increase in the content of hard segments, which leads to an improvement in the compression properties [37]. Recycled polyurethane-based polyols have a higher functionality than conventional petroleum-based polyols, which leads to higher crosslink density. The structure of recycled polyurethane foams has short and difficult to move hard segments, which favors compression strength. The higher the recycled polyol content, the more isocyanate groups are required for the reaction, which leads to higher crosslinking density and the generation of more urea groups, and possibly also to stronger diureido groups, thus increasing the content of hard segments in the recycled polyurethane matrix, which is consistent with the IR spectroscopy results. This formation of high hard chain segments and high crosslink density in the polymer molecule determines the compressive strength of the polyurethane. The increase in crosslink density is also the reason why the apparent density of recycled polyurethane foam increases first with the increase in substitution rate [38,39]; however, when the substitution rate is too high, the reactivity of recycled polyol is weaker than that of polyether 4110, and the reaction between the polyol and the isocyanate becomes less and less, and the formation of crosslinked mesh of the polyurethane is not sufficient [40]. At the same time, due to the fact that the complex structure of recycled polyol also leads to the uneven size and structure of the foam pores, the support of the foam internal pore wall is reduced, and the multiple factors lead to a gradual decrease in its compressive strength and apparent density with the excessive increase in substitution rate. Apparent density is an important performance index of rigid polyurethane foam. Rigid polyurethane foam can be used in the fields of refrigerators, underground direct buried pipes, composite panels, and core materials for construction, etc. Different applications require different polyurethane densities [41]. For example, the apparent density of recycled polyurethane with a substitution rate of 10–30% is greater than 30 kg/cm^3^ and up to 51.27 kg/cm^3^.

#### 3.2.3. Analysis of the Thermal Conductivity of Recycled Polyurethane

**Figure 12:**
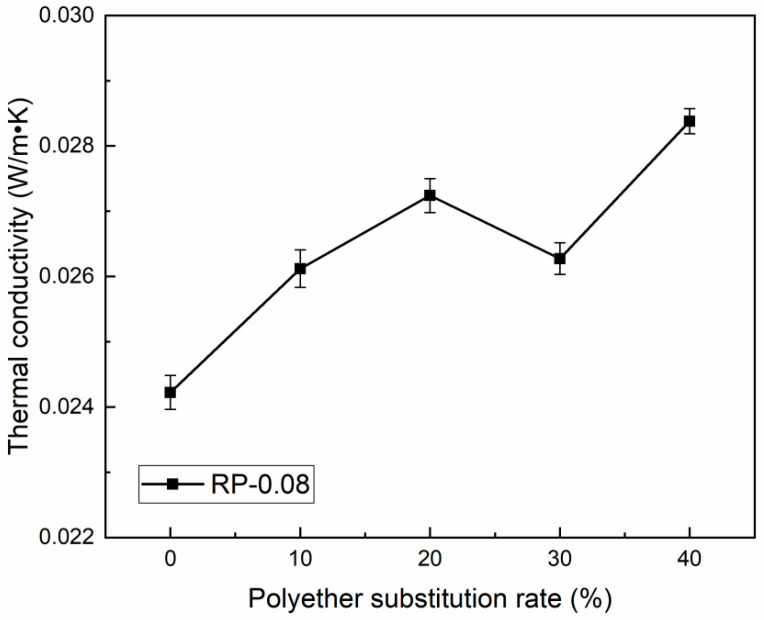
Thermal conductivity of RPU at different substitution rates.

Polyurethane rigid foams are commonly used in insulation applications for which thermal conductivity (λ) is a critical property. The results of thermal conductivity measurements of recycled polyurethane prepared with different substitution rates of recycled polyol RP-0.08 are shown in Figure 12. The thermal conductivity is related to the apparent density of the foam and the thermal conductivity of the gas used as a blowing agent [42]. Although the whole foam contains only a small portion of the polyurethane matrix, denser foams tend to have higher thermal conductivity because their λ values are much higher than those of the blowing agent [43]. Therefore, within a certain range, the thermal conductivity of polyurethane foam gradually increases with the increase in the substitution rate of recycled polyol, and the thermal insulation decreases. When the substitution rate of recovered polyol is 20%, the thermal conductivity increases from 0.0242 W/m·K to 0.0272 W/m·K, which is an increase of 12.40%. When the substitution rate is 30%, the thermal insulation performance of the foam is improved due to the reduction in its apparent density, but the thermal conductivity is also related to the internal structure of the material. When the substitution rate continues to increase, the small density tends to make the tiny window pore structure inside the polyurethane become larger or connected, which leads to the enhancement of the radiation effect of the air between the holes and the convection inside the holes, resulting in the increase in the coefficient of the thermal conductivity of the polyurethane material [44].

#### 3.2.4. Scanning Electron Microscopy Analysis of Recycled Polyurethane

**Figure 13:**
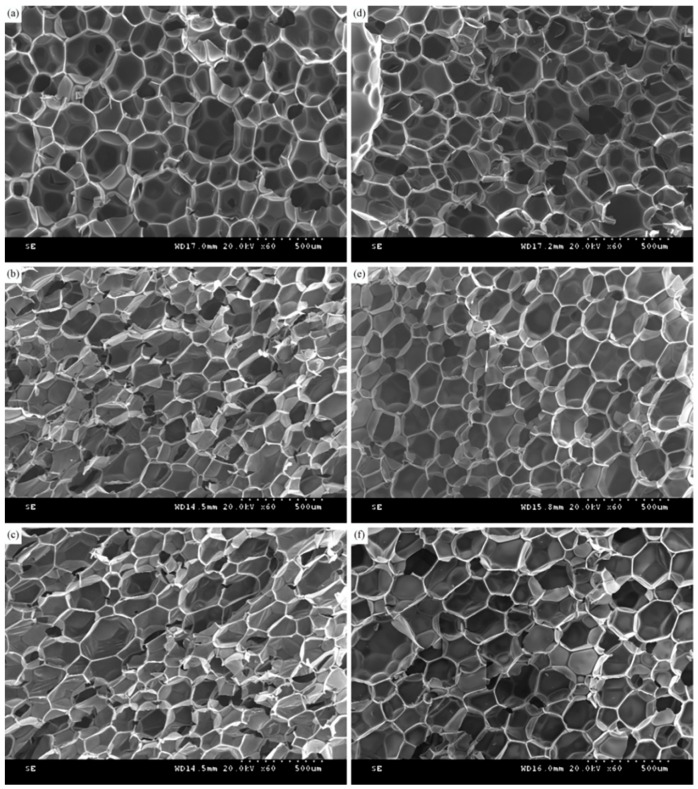
SEM images of RPU prepared with different recycled polyols at a substitution rate of 20%. Types of polyols used: (**a**) Polyether 4110 (**b**) RP-0.02 (**c**) RP-0.04 (**d**) RP-0.06 (**e**) RP-0.08 (**f**) RP-0.10.

Firstly, the microstructure of the polyols was observed, and the SEM images of the recycled polyurethane foams prepared with the recycled polyols RP-0.02, RP-0.04, RP-0.06, RP-0.08, and RP-0.10 obtained at different PN dosages with a 20% substitution rate, respectively, are shown in Figure 13, and most of the polyurethane foams’ bubble pores are pentagonal and hexagonal [45,46]. As can be seen from Figure 13a, the vesicle size of the polyurethane foam RPU-0 prepared from pure polyether 4110 is uniform, with a small number of large pores present, but the vesicles are fine and arranged in a regular and compact manner, and, thus, exhibit high compressive strength. When the amount of the PN catalyst is too small, the polyurethane molecular chain breakage is incomplete, the degradation reaction is not sufficient, which is unfavorable for the application of recycled polyols, and the dimensions of the vesicles of prepared polyurethanes become non-uniform, the vesicles become larger in different degrees, and the phenomenon of foam rupture occurs. As can be seen from Figure 13d–f, with the increase in PN catalyst dosage, the structure of the foam pores of the recycled polyurethane foams was improved, and the skeleton was more regular. The pore structure of PU4 and PU5 was better than that of the pure sample RPU-0. The integrity of the polyurethane foam pores prepared from recycled polyol at a 0.08% PN dosage was better and the shape of the pores was more regular. Polyurethane foams prepared with 20% of polyether 4110 using recycled polyol had a smaller cell size and denser foam structure, indicating better mechanical properties.

**Figure 14:**
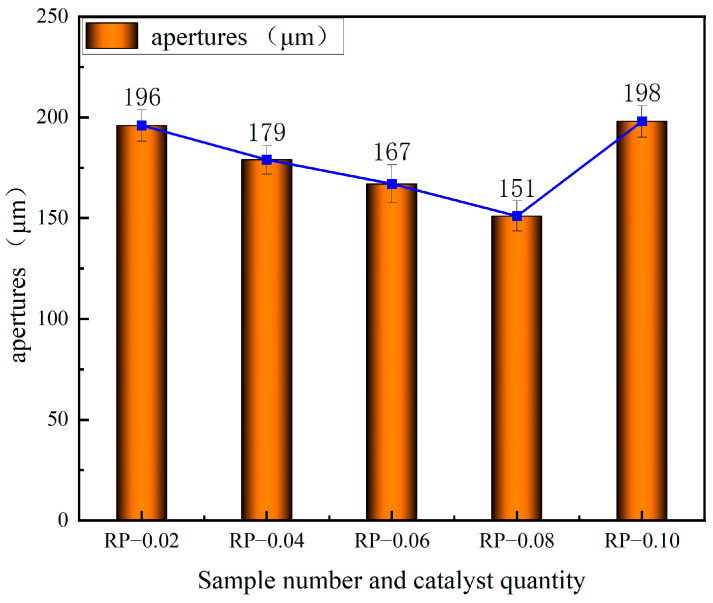
The relationship between the amount of PN catalyst and the size of the pore diameter.

The relationship between the amount of PN catalyst and the pore size is shown in Figure 14.

Types of polyols used: (a) polyether 4110, (b) RP-0.02, (c) RP-0.04, (d) RP-0.06, (e) RP-0.08, and (f) RP-0.10.

**Figure 15:**
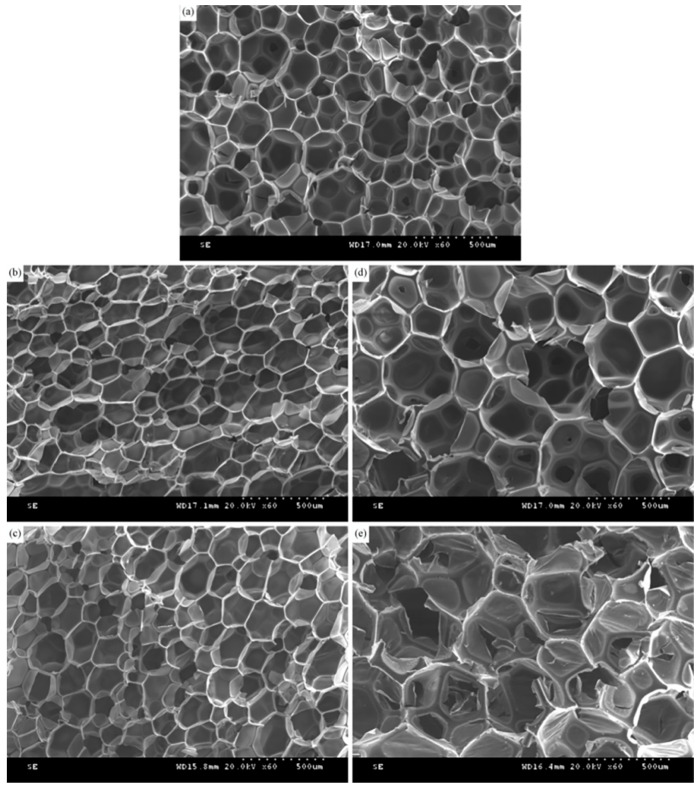
SEM images of recycled polyurethane prepared by recycled polyol RP4 with different substitution rates: (**a**)RPU-0; (**b**) RPU-10; (**c**) RPU-20; (**d**) RPU-30; (**e**) RPU-40.

Figure 15 shows the scanning electron microscope images of polyurethane foams prepared from recycled polyol RP-0.08 at different substitution rates (0%, 10%, 20%, 30%, 40%). For the polyurethane foam RPU-0 prepared from pure polyether 4110, the pores are relatively small, relatively uniformly distributed, and have a certain degree of regularity [47,48,49]. With the increase in the substitution rate, the pore size of the regenerated polyurethane foam first decreases and then increases. When the substitution rate is 20%, the polyurethane foam has more closed pores, a more complete pore film, and a more solid skeleton, which reflects that the foam has a better branched crosslinked structure and good support, with a small window pore and a high closed pore rate. The polyurethane foam pores are tightly arranged and the skeleton is stout, with an excellent supporting effect, so the compression strength of the foam is improved. With the gradual increase in the substitution rate, the pore size of the foam gradually increases, and the distribution of the foam pores is uneven. When the substitution rate of recycled polyol is 40%, the foam pores and skeleton are deformed and crushed to different degrees, and the size of the foam pores is obviously larger, which greatly reduces the thermal insulation performance and compression strength of the foam.

**Figure 16:**
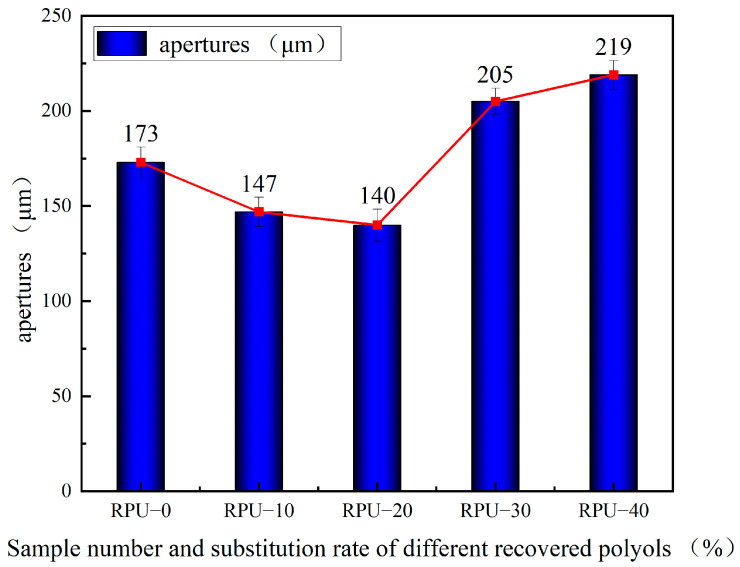
Relationship between different substitution rates and the pore size of recovered polyols.

The relationship between the different substitution rates of recovered polyols and pore size is shown in Figure 16.

#### 3.2.5. Heat Loss Analysis of Recycled Polyurethane

**Figure 17:**
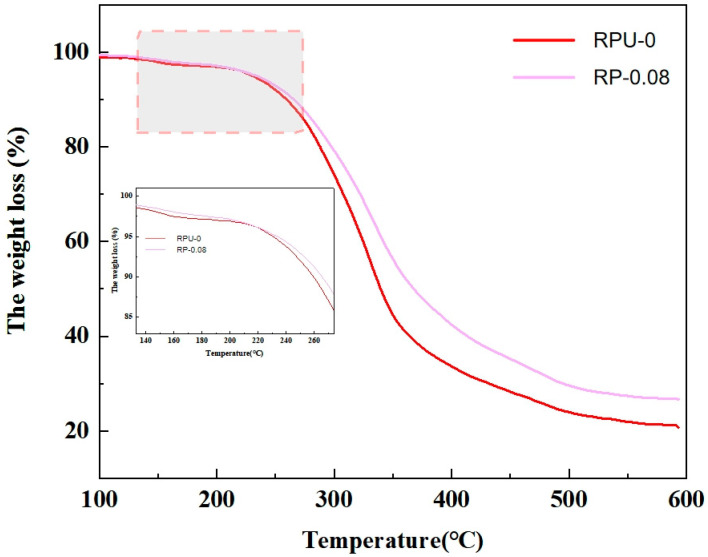
Heat loss curve of recycled polyurethane.

Combining the above conditions of density, compressive strength, and other conditions for the elimination of the prepared foam selection, it was determined that the PN catalyst addition dosage of 0.08% for the better performance of the RP-0.08 foam body and pure samples of RPU-0 thermogravimetric analysis was carried out as shown in Figure 17. From the figure, it can be seen that the phosphorus and nitrogen elements of the PN catalyst have obvious effects on the thermal weight loss analysis curve of polyurethane, which are mainly reflected in four aspects, such as the increase in decomposition temperature, the decrease in decomposition rate, the enhancement of thermal stability, and the change in the morphology of the thermal weight loss curve. The heat weight loss of the two different ratios of foams can be divided into three stages: the first stage is 100–200 °C, where the volatilization of free and bound water in the foam leads to the weight loss of the foam; the second stage is 200–390 °C, which is the isocyanate in the polyurethane linkage with the hard section of the polyurethane break; and the third stage is 390–590 °C, which is the polyether in the polyurethane tip of the polyurethane soft satin break. The substantial weight loss temperature of the foams prepared in two ratios is around 230 °C, and the decomposition stops around 590 °C. The fastest weight loss temperature of RPU-0 is around 327 °C, while the fastest weight loss temperature of RP-0.08 is around 348 °C. It can be clearly seen that, in the area of the local zoomed-in picture, the weight loss of RP-0.08 is obviously more rapid than that of RP-0.08, which suggests that the phosphorus of PN catalyst nitrogen in polyurethane, which is influenced by the elements phosphorus and nitrogen, undergoes a series of complex chemical changes at high temperatures. These changes include the oxidation of the phosphorus element and the nitrogenization of the nitrogen element or participation in the formation of the carbon layer. These chemical changes work together to affect the thermal decomposition process of polyurethane, thus affecting its heat loss curve and forming compounds with high thermal stability, which can slow down the thermal decomposition process of polyurethane. In addition to the above chemical changes, phosphorus and nitrogen elements may also cause changes in the physical structure of polyurethane. As a crosslinking point or reinforcing agent, phosphorus and nitrogen elements are able to form a crosslinking structure and improve the crosslinking density and mechanical strength of polyurethane. These changes in physical structure may also have an effect on the heat loss curve of polyurethane, resulting in a lower rate of the decomposition of polyurethane. This makes it more stable and less likely to decompose. In addition, phosphorus and nitrogen elements may slow down the thermal decomposition process by forming a carbon layer, further reducing the decomposition rate. After the introduction of phosphorus and nitrogen elements, the heat loss curve of polyurethane may undergo morphological changes. It can be seen that the slope of the curve may become flatter, indicating a slower decomposition process; phosphorus and nitrogen elements can significantly improve the thermal stability of polyurethane. Due to the increase in decomposition temperature and the decrease in decomposition rate, the polyurethane can only undergo significant thermal decomposition at higher temperatures, thus exhibiting better thermal stability, which improves the quality of the recycled product and extends its service life. Therefore, the chemical stability of the foam prepared by RP-0.08 is greater than that of RPU-0; thus, it can be shown that the foam prepared by RP-0.08 has good thermal stability and is more stable than RPU-0 [50].

## 4. Conclusions

In this paper, the degradation of waste polyurethane was carried out by using a PN catalyst in synergy with a polyol solvent, testing and characterizing the catalyst and the regenerated polyether polyol after degradation, optimizing the synthesis process, successfully recovering and recycling the polyurethane waste, and preparing regenerated polyurethane materials. The details are as follows:

(1) Waste PU was successfully degraded with a solid–liquid mass ratio of 1:1.5 by using diol as the alcoholysis agent and a PN catalyst, and the changing rules of hydroxyl value, viscosity, and the molecular weight of polyols recovered from degradation products were obtained under different dosages of metal-free PN catalyst through the influence of factors such as reaction temperature and time on the hydroxyl value and the viscosity of degradation products. The optimal experimental conditions were determined to be a reaction temperature of 180 °C, a reaction time of 3 h, a PN dosage of 0.08%, a polyol viscosity of 3716 mPa·s, a hydroxyl value of 409.2 mgKOH/g, and an average molecular weight of 2616, etc. The tests of FTIR, ^1^H, NMR, and other tests showed that the waste polyurethanes were degraded into oligomers successfully, and the regenerated polyether polyol was obtained.
(2) A series of recycled polyurethane materials with different substitution rates were prepared by replacing part of polyether 4110 with recycled polyol RP-0.08 obtained under optimal conditions, which were characterized by FTIR, SEM, compression strength, and thermal conductivity tests, and the results showed that the recycled polyurethane foams possessed good physical properties such as compression strength and apparent density. Within a certain range, the compressive strength of the regenerated polyurethane foam increased from 0.272 MPa at the original (substitution rate of 0%) to a maximum value of 0.390 MPa (substitution rate of 20%) with the increase in the substitution rate, which was an increase of 43.38%. When the substitution rate is 20%, the thermal conductivity of the foam is increased from 0.0242 W/m·K to 0.0272 W/m·K, which is increased by 12.40%, although the thermal insulation performance is reduced, but it is maintained within a certain range, and it also has a certain degree of thermal insulation.
(3) The vesicles of polyurethane showed different degrees of changes after the addition of different recycled polyols, and the SEM images showed that the increase in the amount of PN helped to improve the vesicle structure of the polyurethane foam formed by the addition of recycled polyol. When the recycled polyol RP-0.08 was used to prepare recycled polyurethane with 10% and 20% substitution rates, it helped to improve the microstructure of polyurethane foam, which had a good vesicle skeleton with uniform vesicle size and regular arrangement, reflecting good compression properties. When the substitution rate continues to increase, the pores gradually become larger and even rupture.

## Figures and Tables

**Table 1:** The formula of regenerated polyurethane material.

Reagents	Mass (g)	Percentage (%, Based on Total Polyols)
Polyether polyol 4110	30, 28.5, 27, 24, 21, 18	100, 95, 90, 80, 70, 60
Recycled polyol RP-0.08	0, 1.5, 3, 6, 9, 12	0, 5, 10, 20, 30, 40
Dimethylsiloxane	0.54	1.80
Dichloroethane monofluoride	7.5	25.0
Triethanolamine	0.45	1.50
Dibutyltin dilaurate	0.15	0.50
Isocyanate PM200	36	120

**Table 2:** Number of average molecular weight and polymer dispersity index of recovered polyols under different PN catalyst dosages.

Sample Number	PN Catalyst Usage/%	Mn	PDI
RP-0.02	0.02	4804	1.468
RP-0.04	0.04	3769	1.403
RP-0.06	0.06	3224	1.322
RP-0.08	0.08	2616	1.214
RP-0.10	0.10	2465	1.217
Polyether 4110	--	1104	1.179

## Data Availability

The original contributions presented in the study are included in the article, further inquiries can be directed to the corresponding authors. Data are contained within the article.
